# Activated human mesenchymal stem/stromal cells suppress metastatic features of MDA-MB-231 cells by secreting IFN-*β*

**DOI:** 10.1038/cddis.2016.90

**Published:** 2016-04-14

**Authors:** N Yoon, M S Park, T Shigemoto, G Peltier, R H Lee

**Affiliations:** 1 Texas A&M University Health Science Center, College of Medicine, Institute for Regenerative Medicine, Temple, TX, USA

## Abstract

Our recent study showed that human mesenchymal stem/stromal cells (hMSCs) are activated to express tumor necrosis factor (TNF)-*α*-related apoptosis-inducing ligand (TRAIL) by exposure to TNF-*α* and these activated hMSCs effectively induce apoptosis in triple-negative breast cancer MDA-MB-231 (MDA) cells *in vitro* and *in vivo*. Here, we further demonstrated that activated hMSCs not only induced apoptosis of MDA cells but also reduced metastatic features in MDA cells. These activated hMSC-exposed MDA cells showed reduced tumorigenicity and suppressed formation of lung metastasis when implanted in the mammary fat pad. Surprisingly, the activated hMSC-exposed MDA cells increased TRAIL expression, resulting in apoptosis in MDA cells. Interestingly, upregulation of TRAIL in MDA cells was mediated by interferon-beta (IFN-*β*) secreted from activated hMSCs. Furthermore, IFN-*β* in activated hMSCs was induced by RNA and DNA released from apoptotic MDA cells in absent in melanoma 2 (AIM2) and IFN induced with helicase C domain 1 (IFIH1)-dependent manners. These observations were only seen in the TRAIL-sensitive breast cancer cell lines but not in the TRAIL-resistant breast cancer cell lines. Consistent with these results, Kaplan–Meier survival analysis also showed that lack of innate sensors detecting DNA or RNA is strongly associated with poor survival in estrogen receptor-negative breast cancer patients. In addition, cancer-associated fibroblasts (CAF) isolated from a breast cancer patient were also able to express TRAIL and IFN-*β* upon DNA and RNA stimulation. Therefore, our results suggest that the crosstalk between TRAIL-sensitive cancer cells and stromal cells creates a tumor-suppressive microenvironment and further provide a novel therapeutic approach to target stromal cells within cancer microenvironment for TRAIL sensitive cancer treatment.

Mesenchymal stem/stromal cells (MSCs) have been investigated extensively for cancer treatment because of their excellent homing ability to the tumor.^1, 2, 3^ However, the previous studies showed controversial results and it still remains unclear whether MSCs promote or suppress tumor progression. Many studies have shown that MSCs show pro-tumorigenic effects by promoting proliferation of a cancer-initiating population^4, 5, 6, 7^ or stimulate metastasis^8, 9, 10^ by secreting pro-tumorigenic cytokines or through crosstalk with cancer cells. Furthermore, recent studies showed that tumors recruit MSCs and induce their conversion into cancer-associated fibroblasts (CAFs)^11, 12, 13^ that are associated with tumor progression,^14, 15, 16, 17^ invasion and metastasis,^16, 17, 18, 19^ therapeutic resistance^15, 20, 21^ and prognosis in breast cancer.^22^

Our recent study demonstrated that human MSCs (hMSCs) are able to express the high level of an apoptosis-inducing factor, tumor necrosis factor (TNF)-*α*-related apoptosis-inducing ligand (TRAIL) upon TNF-*α* stimulation and induce apoptosis in triple-negative breast cancer cell (TNBC) lines including MDA-MB-231 (MDA) cells.^23^ Interestingly, TRAIL expression in hMSCs is further increased by stimulation of DNA and RNA released from apoptotic MDA cells and such antitumorigenic effect of hMSCs is only shown in TRAIL-sensitive TNBC lines.^23, 24^ These results suggest that the crosstalk between hMSCs and cancer cells may differ depending on the types of cancer, and further study is required to examine whether the crosstalk between TRAIL-expressing activated hMSCs and TRAIL-sensitive cancer cells creates a tumor-suppressive environment and thereby further suppresses tumor progression.

In this study, we examined effects of activated hMSCs on metastatic features of MDA cells. Our results showed that the crosstalk between TRAIL-expressing activated hMSCs and TRAIL-sensitive cancer cells not only induced apoptosis of cancer cells but also reduced metastatic features of MDA cells, which was mediated by the hMSC-derived interferon-beta (IFN-*β*).

## Results

### MDA cells decrease metastatic features after coculture with TNF-*α* activated hMSCs

**Figure 1:**
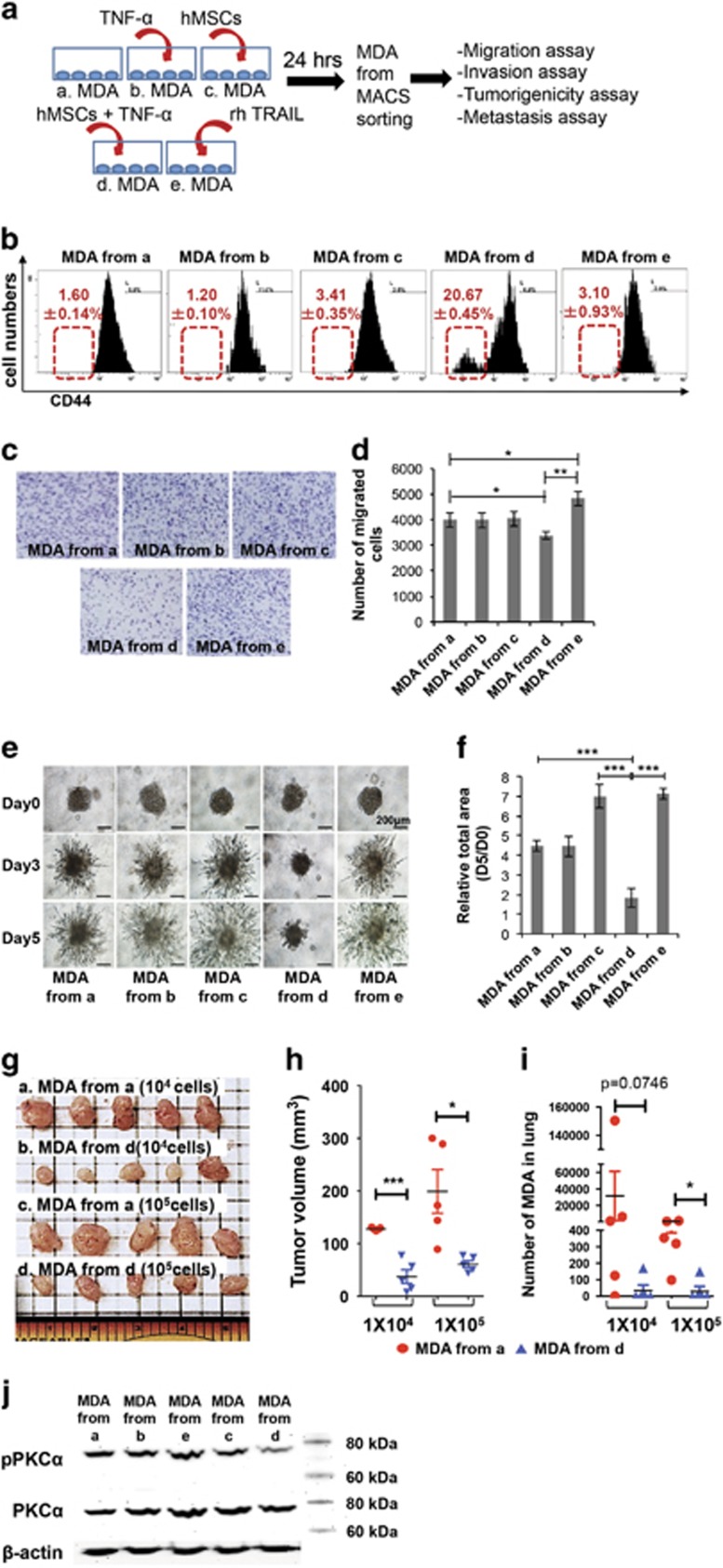
MDA cells lose their metastatic ability upon coculture with activated hMSCs. (**a**) Schematic diagram. (**b**) Representative images from flow cytometry analyses detecting CD44 expression in MDA cells under different conditions. Values are mean±S.D. *n*=3. (**c** and **d**) Cell migration assay; representative images (**c**) and the counted number of migrated MDA cells (**d**) of migration assay. Values are mean±S.D. *n*=4; *P<0.05; **P<0.01; one-way ANOVA. (**e** and **f**) Invasion assay of MDA cells; representative images (**e**) and quantified total area at day 5 (**f**) of 3D invasion assay. Values are mean±S.D. *n*=6; **P<0.01; *** *P*<0.001; one-way ANOVA. (**g** and **h**) Images (**g**) and calculated volume (**h**) of primary tumors in the mammary fat pad of mice 6 weeks after implantation of MDA cells as shown in Figure 1a. *n*=5; *P<0.05; ***P<0.001; Student's *t*-test. (**i**) Metastasized MDA cells in lungs of animals from (**g**) using quantitative PCR for human Alu sequences. *n*=5; *P<0.05; Student's *t*-test. (**j**) Western blot analysis for PKC-*α* expression in MDA cells isolated from different conditions as indicated in Figure 1a

The metastatic cancer features that are characterized by high invasiveness, tumorigenicity, metastatic potential and drug resistance are closely associated with poor prognosis in several types of cancer.^25^ From our previous study, we demonstrated that TNF-*α*-activated hMSCs (act hMSCs) induce apoptosis in TRAIL-sensitive cancer cells.^23^ To further examine effects of act hMSCs on cancer cells, the metastatic features were analyzed in MDA cells after treatments as shown in Figure 1a. The remaining MDA cells that were isolated by magnetic-activated cell sorting (MACS) negative using CD90 (Supplementary Figure S1), a marker of hMSCs, showed the decreased expression of CD44 (Figure 1b) – a marker for cancer-initiating cells.^26, 27^ In addition, MDA cells exhibited less migration and invasive properties after coculture with act hMSCs (Figures 1c–f). Unlike act hMSC coculture, the MDA cells from coculture with naive hMSCs (CC) or treatment of recombinant human TRAIL (rhTRAIL) increased invasion compared with the control MDA cells (Figures 1c–f). To examine tumorigenic properties of MDA cells after act hMSC coculture, the live MDA cells after act hMSC coculture were implanted into mouse mammary fat pad for the tumor burden formation. After 6 weeks of implantation, the sizes of tumor burdens formed by MDA cells from coculture were significantly smaller than naive MDA cells (Figures 1g and h). We also collected lungs of these animals to see whether any of the MDA cells metastasize from their primary tumor sites to the lung. Detection of human Alu sequences using quantitative PCR of genomic DNA of the lungs^28^ showed that only very few or no human cells were detected in the lung of animals received MDA cells isolated from coculture with act hMSCs, whereas the animals received control MDA cells showed significant numbers of metastasized cells in lungs (Figure 1i). The live MDA cells after act hMSC coculture showed decreased expression of protein kinase C-alpha (PKC-*α*), whereas rhTRAIL treatment increased PKC-*α* (Figure 1j), which is highly expressed in metastatic cancer cells.^29, 30, 31^ These data suggest that act hMSCs not only induce cancer cell death but also suppress metastatic features of MDA cells through coculture.

### Act hMSCs induce apoptosis in rhTRAIL-resistant MDA cells

**Figure 2:**
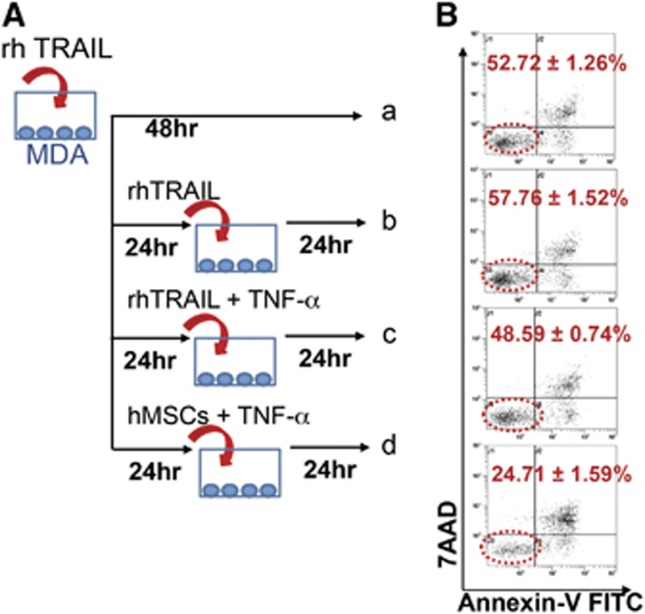
Activated hMSCs induce apoptosis in rhTRAIL-resistant MDA cells. (**A**) Schematic diagram. a: MDA cells treated with rhTRAIL once and cultured for 48 h; b: MDA cells treated with rhTRAIL every 24 h; c: MDA cells treated with rhTRAIL at 0 h followed by rhTRAIL and TNF-α at 24 h; d: MDA cells treated with rhTRAIL at 0 h followed by hMSCs and TNF-α at 24 h. (**B**) Flow cytometry analysis for MDA cells apoptosis after sorting from control MDA cells or coculture with activated hMSCs. Values are mean±S.D. *n*=3

To examine the effect of act hMSCs on resistance to TRAIL-induced apoptosis in MDA cells, we treated MDA cells with rhTRAIL or act hMSCs as shown in Figure 2A. Consistent with the previous observations,^32, 33, 34^ rhTRAIL-exposed MDA cells exhibited less sensitivity to the second treatment of rhTRAIL (Figure 2B(b)). We considered these MDA cells as rhTRAIL-resistant cells. To investigate whether activated hMSCs are able to induce cell death in rhTRAIL-resistant MDA cells, these MDA cells were cocultured with act hMSCs (Figure 2B(d)). The act hMSCs induced >70% of cell death in the rhTRAIL-resistant MDA cells (Figure 2B(d)). As a control, we treated the rhTRAIL-resistant MDA cells with TNF-*α*, which did not induce any further apoptosis (Figure 2B(c)). These observations suggest that the TRAIL-expressing act hMSCs further induce apoptosis in rhTRAIL-resistant MDA cells.

### IFN-*β* induces TRAIL upregulation in MDA cells during coculture with act hMSCs

**Figure 3:**
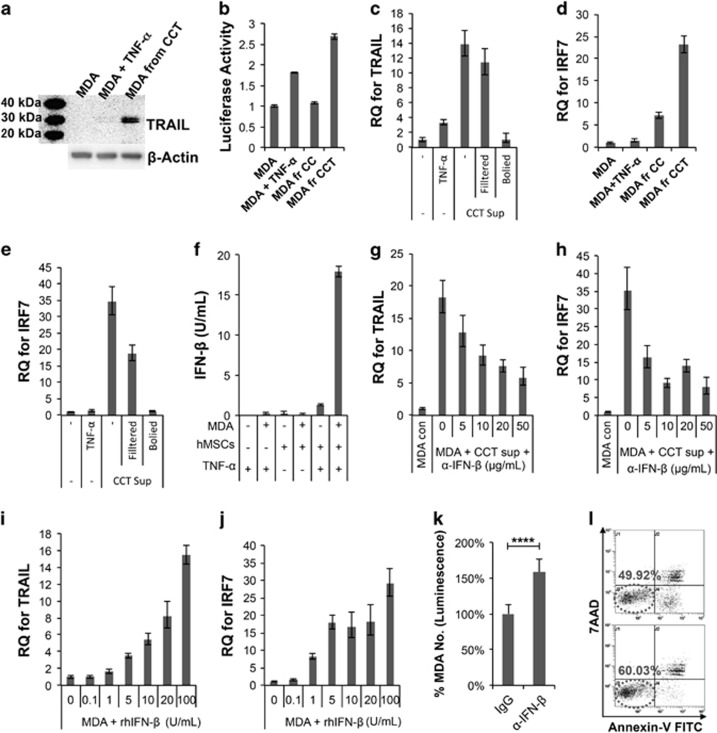
TRAIL is upregulated in MDA cells, following coculture with activated hMSCs. (**a**) Western blot analysis of TRAIL expression in MDA cells from control, TNF-*α* (20 ng/ml) treatment or coculture with act hMSCs (MDA+hMSCs+TNF-*α*; CCT). (**b**) Luciferase assay detecting TRAIL promoter activity in TRAIL luciferase-expressing MDA cells with following conditions; control, TNF-*α* (20 ng/ml) treatment, CC or CCT for 24 h. Values are mean±S.D. (**c**) Quantitative RT-PCR for TRAIL from MDA cells from control, TNF-*α* (20 ng/ml) treatment or treated with supernatant of MDA+hMSC+TNF-*α* coculture (CCT sup). CCT sups were used either as it is (−), filtered with 0.2 *μ*m syringe filter (Filtered) or boiled at 95 °C for 5 min (Boiled). Values are mean±S.D. for triplicate of the assay. (**d**) Quantitative RT-PCR for IRF7 from MDA cells from control, TNF-*α* (20 ng/ml) treatment or coculture with hMSCs (MDA+hMSCs; CC) or act hMSCs (MDA+hMSCs+TNF-*α*; CCT). Values are mean±S.D. for triplicate of the assay. (**e**) Quantitative RT-PCR for IRF7 from MDA cells treated as in (**c**). Values are mean±S.D. for triplicate of the assay. (**f**) ELISA assay for IFN-*β* from the supernatant of MDA and hMSC coculture. Values are mean±S.D. for octuplicate of the assay. (**g** and **h**) Quantitative RT-PCR for TRAIL (**g**) and IRF7 (**h**) from MDA cells from control or treated with supernatant of MDA+hMSC+TNF-*α* coculture (CCT sup) with different concentrations of IFN-*β*-neutralizing antibody (*α*-IFN-*β*; R&D Systems). Values are mean±S.D. for triplicate of the assay. (**i** and **j**) Quantitative RT-PCR for TRAIL (**i**) and IRF7 (**j**) from MDA cells treated with IFN-*β* recombinant protein (rhIFN-*β*). Values are mean±S.D. for triplicate of the assay. (**k**) Luciferase assay of MDA cells, following coculture with activated hMSCs and IFN-*β*-neutralizing antibody (*α*-IFN-*β*; 20 *μ*g/ml). Values are mean±S.D. *n*=6; ****P<0.0001; Student's *t*-test. (**l**) Flow cytometry analysis of apoptosis in MDA cells, following coculture with activated hMSCs and IFN-*β*-neutralizing antibody (*α*-IFN-*β*; 20 *μ*g/ml)

Surprisingly, we found that MDA cells also expressed the TRAIL protein, following coculture with act hMSCs. Western blot analysis showed that MDA isolated from coculture with act hMSCs also expressed a high level of TRAIL protein (Figure 3a). TRAIL luciferase reporter assay also confirmed the upregulation of TRAIL expression at the transcriptional level in cancer cells during coculture with act hMSCs (Figure 3b). To find out if a soluble factor induces TRAIL upregulation in MDA cells, MDA cells were treated with the conditioned media derived from act hMSC-MDA coculture (CCT sup). TRAIL expression was upregulated in MDA cells upon conditioned media treatment, and the upregulation was negated when the conditioned media was boiled before treatment, suggesting soluble factors in the conditioned media may induce TRAIL upregulation (Figure 3c).

In a previous study, it was shown that TRAIL level is regulated by transcription factors related to IFNs, called IFN-related factors (IRFs).^35^ Among several IRFs, Huang, *et al.* found that IRF1 and IRF7 upregulate TRAIL expression in macrophages upon HIV infection. We also found that IRF7 levels in MDA cells, upon coculture with act hMSCs, were upregulated markedly (Figure 3d). The upregulation of IRF7 was also observed in the MDA cells treated with act hMSC-MDA coculture conditioned media but not with boiled conditioned media (Figure 3e). It has been shown that the transcription factor IRF7 is essential for the induction of IFN-*α*/*β* genes,^36^ thus, the levels of IFN-*β* were assessed in conditioned media of coculture. Our result showed that IFN-*β* was detected only in the conditioned media from act hMSC-MDA coculture (Figure 3f). When IFN-*β* was blocked using a neutralizing antibody in the conditioned media, TRAIL upregulation was negated in MDA cells (Figure 3g) as well as the expression level of IRF7 (Figure 3h). Conversely, expression levels of TRAIL and IRF7 were increased in a dose-dependent manner upon recombinant human IFN-*β* (rhIFN-*β*) treatment in MDA cells (Figures 3i and j). In addition, blocking IFN-*β* during coculture with act hMSCs suppressed apoptosis significantly in MDA cells (Figures 3k and l). These results showed that MDA cells also expressed TRAIL during coculture with act hMSCs and the TRAIL expression in MDA cells was mediated by IFN-*β* in conditioned media.

### DNA/RNA fragments from apoptotic cells induce secretion of IFN-*β* in act hMSCs

**Figure 4:**
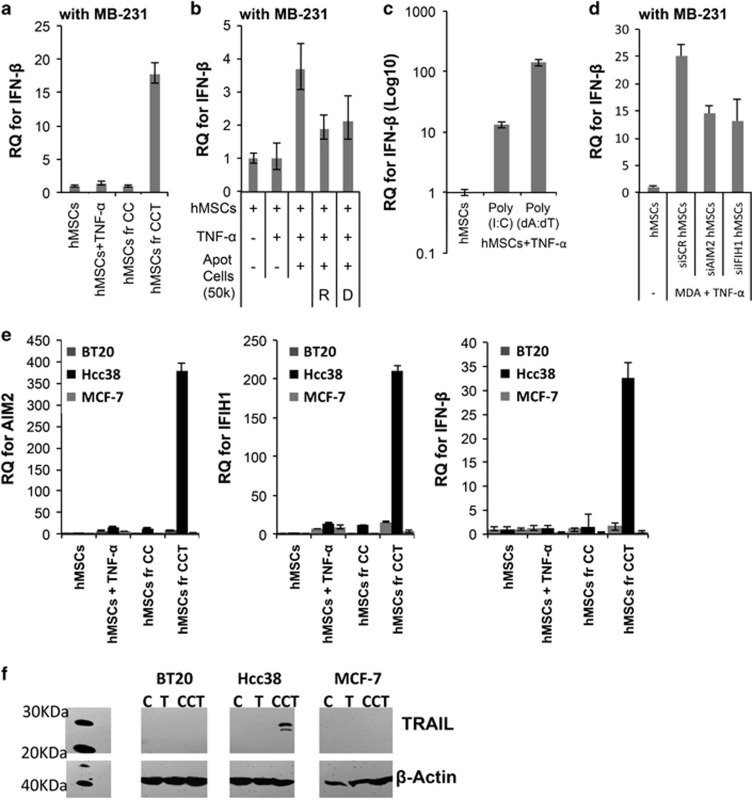
IFN-*β* is derived from activated hMSCs, following coculture with MDA cells. (**a**) Quantitative RT-PCR for IFN-*β* from hMSCs after coculture with MDA (MB-231) cells. Values are mean±S.D. for triplicate of the assay. (**b**) Real-time RT-PCR for IFN-*β* from hMSCs treated with apoptotic MDA (MB-231) cells (Apot cells). Apoptotic MDA cells were treated with either RNase (R) or DNase (D). Values are mean±S.D. for triplicate of the assay. (**c**) Quantitative RT-PCR for IFN-*β* from hMSCs treated with TNF-*α* and either poly(I:C) or poly(dA:dT) (1 *μ*g/ml). Values are mean±S.D. for triplicate of the assay. (**d**) Quantitative RT-PCR for IFN-*β* from hMSCs after coculture with MDA (MB-231) cells. Before coculture, hMSCs were treated with siRNA for AIM2 (siAIM2 hMSCs) or IFIH1 (siIFIH1 hMSCs). Scrambled siRNA (siSCR hMSCs) were used as a control. Values are mean±S.D. for triplicate of the assay. (**e**) Quantitative RT-PCR assays for AIM2, IFIH1 and IFN-*β* in hMSCs, following coculture with Hcc38, MCF-7 and BT20 cells for 24 h. CC, hMSCs+cancer cells; CCT, hMSCs+cancer cells+TNF-*α*. Values are mean±S.D. for triplicate of the assay. (**f**) Western blot assay for TRAIL expression in BT20, Hcc38 and MCF-7 cells from control (C), TNF-*α* (T; 20 ng/ml) treatment or coculture with act hMSCs (MDA+hMSCs+TNF-*α*; CCT)

To examine the source of IFN-*β* in the conditioned media of coculture, hMSCs, and MDA cells were separated following 24-h coculture using MACS sorting. Real-time RT-PCR showed that IFN-*β* level was upregulated in act hMSCs after coculture (Figure 4a). The IFN-*β* was not detected in MDA cells in any conditions (data not shown), suggesting that IFN-*β* is mainly secreted from act hMSCs.

Type I IFNs are well-known cytokines to be produced in many types of cells in response to viral infection.^37^ During the innate immune response, cytosolic DNA/RNA fragments are detected as danger signals, activating several receptors and induce production of cytokines such as IFN-*β*.^38, 39^ Previously, we have also shown that RNA/DNA fragments from apoptotic cells triggers innate sensors (ISs) such as absent in melanoma 2 (AIM2) and IFN induced with helicase C domain 1 (IFIH1) in hMSCs.^24^ Here, we also observed that IFN-*β* was induced when hMSCs were treated with apoptotic MDA cells (Figure 4b). Messenger RNA level of IFN-*β* was also upregulated by apoptotic MDA cell treatment (Figure 4b). However, such upregulation was decreased upon RNase and DNase treatment (Figure 4b). Consistent with this result, IFN-*β* mRNA was upregulated by co-treatment with TNF-*α* and either polyinosinic:polycytidylic acid (poly(I:C)), a synthetic analog of double-stranded RNA, or poly(dA:dT), a repetitive synthetic double-stranded DNA (Figure 4c). IFN-*β* mRNA from hMSCs was increased upon poly(deoxyadenylic-deoxythymidylic) acid (poly(dA:dT)) treatment in a dose-dependent manner (Supplementary Figure S2). To examine whether act hMSCs induce IFN-*β* secretion in an AIM2 or IFIH1-dependent manner, we examined expression levels of IFN-*β* in hMSCs after AIM2 or IFIH1 siRNA transfection. Upon AIM2 or IFIH1 blockage in hMSCs (Supplementary Figure S3), the levels of IFN-*β* were partially decreased (Figure 4d), suggesting DNA/RNA fragments from apoptotic cells induce secretion of IFN-*β* from hMSCs. Upregulation of AIM2, IFIH1 and IFN-*β* was also observed in hMSCs upon coculture with another TRAIL-sensitive cancer cell line, Hcc38 (Figure 4e), which is also sensitive to coculture with act hMSCs.^23, 40^ However, the expression levels of AIM2, IFIH1 and IFN-*β* in hMSCs were not upregulated following coculture with TRAIL-resistant cancer cell lines^40^ (Figure 4e): estrogen receptor (ER)-positive breast cancer MCF-7 cells and TNBC BT20 cells.^40^ Furthermore, TRAIL upregulation upon coculture with hMSCs, as observed in MDA cells, was also observed in additional TRAIL-sensitive cancer cell line, Hcc38, but not in TRAIL-resistant cancer cell lines, BT20 and MCF-7 (Figure 4f). These results demonstrated that the IS-mediated crosstalk is induced only between activated hMSCs and TRAIL sensitive cancer cells, suggesting that crosstalk between hMSCs and cancer cells differs depending on the types of cancer.

AIM2 is one of the factors that are upregulated by IFN-*β*.^41^ We also observed that when hMSCs were treated with rhIFN-*β*, AIM2 level was upregulated markedly (Supplementary Figure S4A). The AIM2 level was also markedly increased in hMSCs upon treatment with the supernatant of the coculture with MDA cells, and such a high level of AIM2 was negated upon IFN-*β*-neutralizing antibody treatment during coculture (Supplementary Figure S4E). The level of IFIH1, the RNA sensor was also upregulated by rhIFN-*β* treatment (Supplementary Figure S4B) and by treatment with the supernatant of the coculture (Supplementary Figure S4F). Like AIM2, IFIH1 level was also low during coculture when IFN-*β*-neutralizing antibody was treated (Supplementary Figure S4F). Like MDA cells, when hMSCs were treated with rhIFN-*β*, the levels of IRF7 and TRAIL were upregulated markedly (Supplementary Figures S4C and S4D), and IFN-*β* antibody negated such upregulation upon treatment with the supernatant of the coculture (Supplementary Figures S4G and S4H). These results suggest that IS-mediated IFN-*β* secretion from hMSCs creates feed-forward stimulation in both hMSCs and MDA cells, and AIM2/IFIH1 ISs and IFN-*β* are co-dependent for antitumorigenic properties of act hMSCs.

### IFN-*β* expressed by hMSCs during coculture is one of the main factors that reduce metastatic features of MDA cells

**Figure 5:**
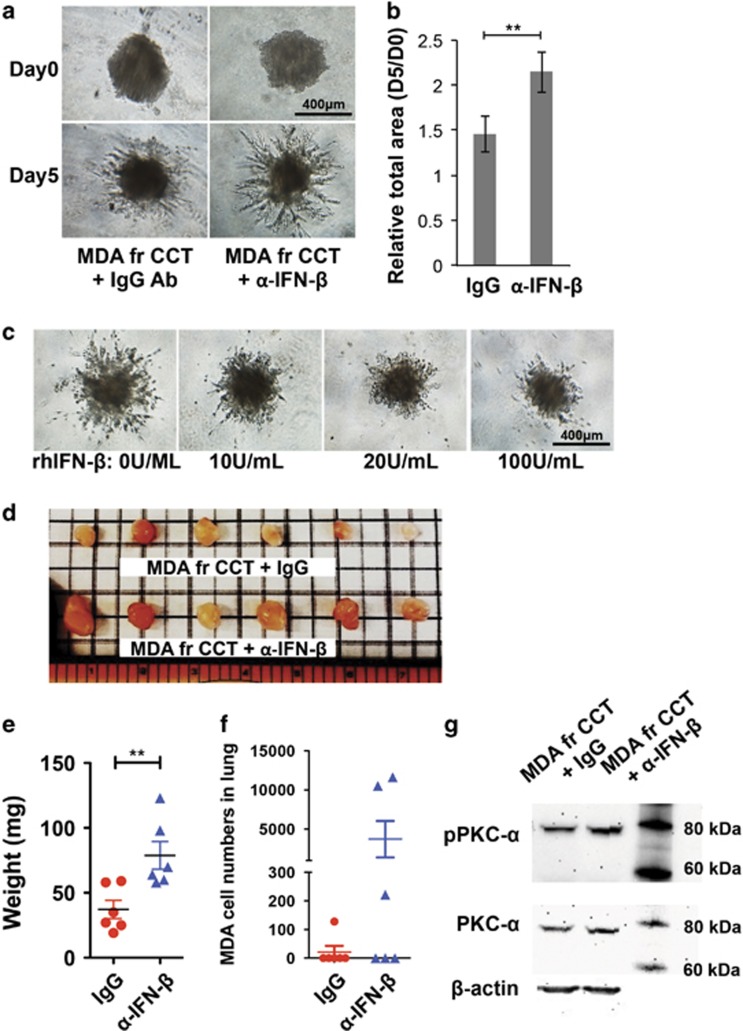
Activated hMSC-derived IFN-*β* suppress tumorigenicity and metastasis of MDA cells. (**a** and **b**) Invasion assay; representative images (**a**) and total area (**b**) of cell invasion assay of MDA cells isolated from act hMSC coculture with IgG or IFN-*β*-neutralizing antibody (*α*-IFN-*β*; 20 *μ*g/ml). (**c**) Representative images of cell invasion assay of MDA cells treated with recombinant human IFN-*β* (rhIFN-*β*; 100 U/ml; R&D Systems). Values are mean±S.D. *n*=6; ***P<0.001; one-way ANOVA. (**d** and **e**) Images (**d**) and weight (**e**) of primary tumors in the mammary fat pad of mice 6 weeks after implantation of MDA cells isolated from act hMSC coculture with IgG or IFN-*β*-neutralizing antibody (*α*-IFN-*β*; 20 *μ*g/ml). *n*=6; **P<0.01; Student's *t*-test. (**f**) Metastasized MDA cells in lungs of animals from (**d**) using quantitative PCR detecting human Alu sequences. *n*=6. (**g**) Western blot analysis of PKC-*α* phosphorylation and expression in MDA cells from act hMSC coculture with IgG or IFN-*β-*neutralizing antibody (*α*-IFN-*β*; 20 *μ*g/ml)

To investigate whether act hMSC-derived IFN-*β* is responsible for the reduced metastatic features of MDA cells as shown in Figure 1, we applied IFN-*β*-neutralizing antibody (*α*-IFN-*β*) during coculture for 24 h and sorted MDA cells for *in vitro* invasion and *in vivo* tumorigenicity assays. The *α*-IFN-*β*-treated MDA cells exhibited more invasive properties than the IgG control antibody (IgG)-treated MDA cells during coculture (Figures 5a and b). When we treated the rhIFN-*β* pre-treated MDA cells for 24 h as a control, the invasion was also reduced in a dose-dependent manner (Figure 5c). Consistent with this result, the reduced tumorigenic properties in MDA cells after coculture with act hMSCs were partially negated by the IFN-*β* blockage during coculture (Figures 5d and e). In addition to becoming more tumorigenic MDA cells after IFN-*β* blockage, metastasized MDA cell numbers in the lungs were also increased by IFN-*β* blockage during coculture (Figure 5f), although there was no statistical significance because of a large variation in the cell numbers. The IFN-*β*-neutralizing antibody-treated MDA cells exhibited an increase in both phosphorylation and expression of PKC-*α* than IgG-treated MDA cells during coculture (Figure 5g). These results suggest that the crosstalk between act hMSCs and MDA cells suppresses invasive and metastatic properties in MDA cells.

### Lack of ISs detecting DNA or RNA is strongly associated with poor survival in ER-negative breast cancer patients

**Figure 6:**
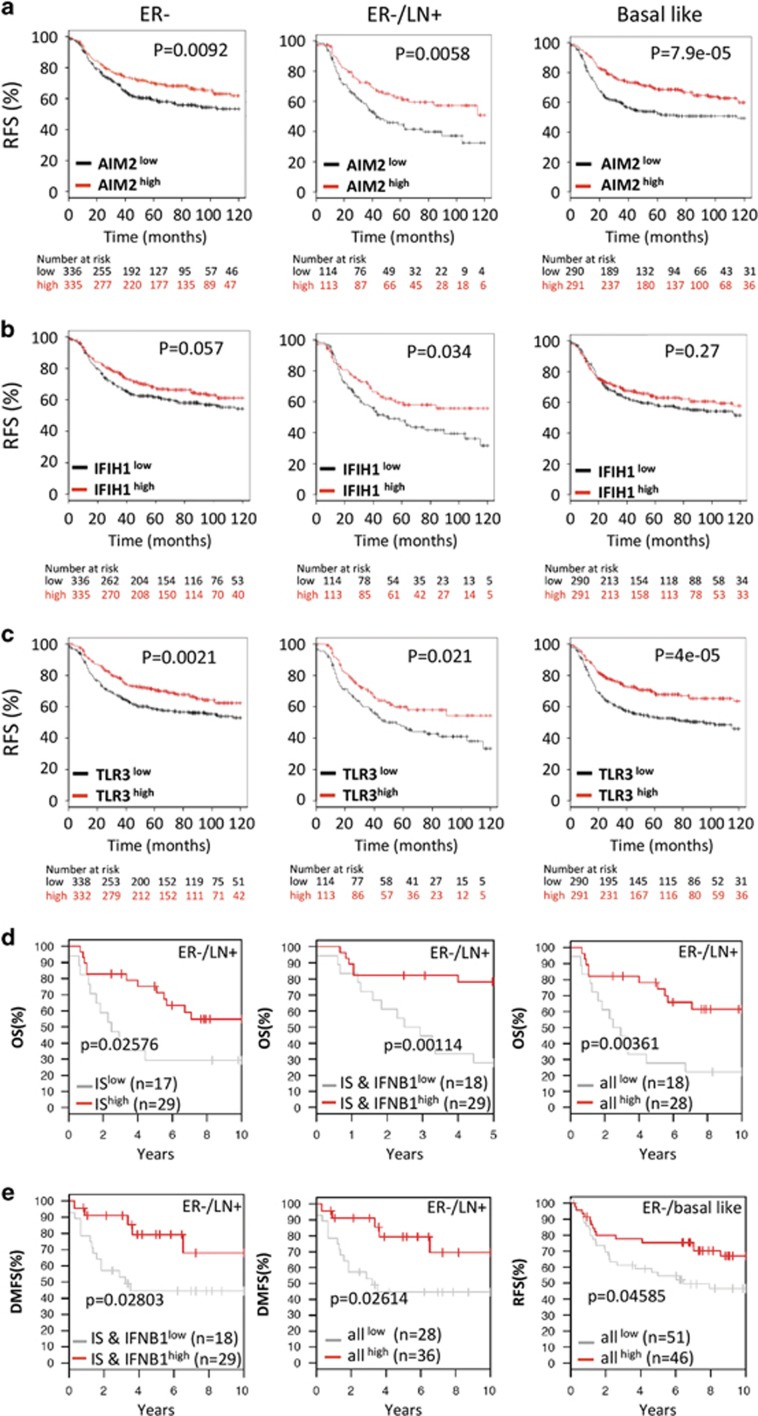
Kaplan–Meier survival analysis of the ISs and related genes in breast cancer. (**a**-**c**) Kaplan–Meier survival analysis of the individual IS in all, ER-positive, ER-negative, ER-negative/lymph node positive and basal-like breast cancer patients. (**d** and **e**) Kaplan–Meier survival analysis of the gene set of ISs (AIM2, IFIH1 and TLR3), IS and IFNB1 and all (IS, IFNB1, IRF7 and TRAIL). Relapse-free survival (RFS); overall survival (OS); distant metastasis-free survival (DMFS)

Numerous studies have investigated the prognostic importance of single genes or sets of genes in breast cancer.^42, 43, 44, 45^ To examine prognostic significance of ISs detecting DNA or RNA in breast cancer patient survival, we analyzed correlations between the expression levels of the gene set (AIM2, IFIH1, Toll-like receptor 3 (TLR3), IRF7, IFN-*β* and/or TRAIL) and breast cancer patient survival rate using Kaplan–Meier survival analysis, and log-rank *P*-values were calculated using the online tools.^46, 47^ Our results showed that lack of these ISs was strongly associated with poor survival in lymph node positive, ER-negative and basal-like breast cancer patients (Figures 6a–c). Consistent with our *in vitro* coculture results with ER-positive MCF-7 cells, there was no correlation between either AIM2 or IFIH1 and ER-positive breast cancer patient survival (Supplementary Figures S4A–4C). Interestingly, the gene sets of ISs (AIM2, IFIH1 and TLR3), ISs and IFN-*β* (IFNB1), and all (ISs, IFNB1, IRF7 and TRAIL) showed strong correlations in ER-negative and lymph node positive or basal-like breast cancer patient survival (Figure 6d and e).

### CAFs are capable of expressing TRAIL and IFN-*β*

**Figure 7:**
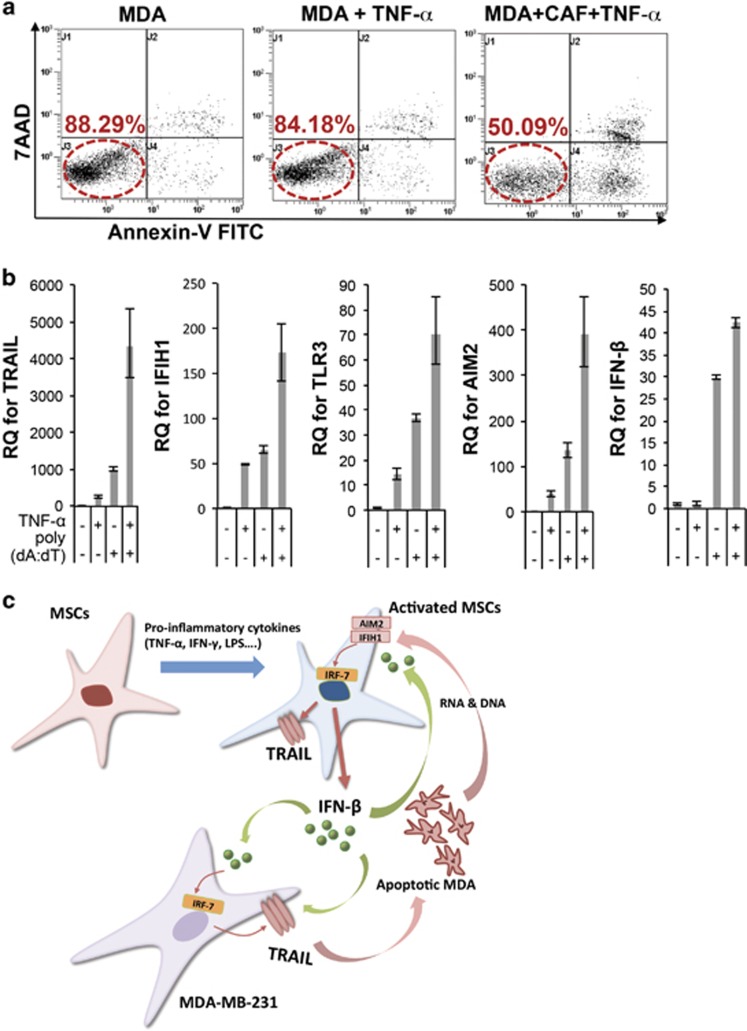
Activated CAFs induce apoptosis in MDA cells and upregulate ISs and IFN-*β* upon poly(dA:dT) stimulation. (**a**) Flow cytometry analysis in MDA cells after coculture with activated CAFs (MDA+CAF+TNF-α). (**b**) Quantitative RT-PCR for AIM2, IFIH1, TLR3, TRAIL and IFN-*β* from CAFs treated with or without TNF-*α* (20 ng/ml) or poly(dA:dT) (1 *μ*g/ml) for 24 h. Values are mean±S.D. for triplicate of the assay. (**c**) Schematic diagram summarizing the observations of this study

Next, we examined whether CAFs are capable of expressing TRAIL and ISs upon TNF-*α* stimulation and inducing IFN-*β* expression upon the stimulation of RNA or DNA released by apoptotic cells. Our results showed that TNF-*α*-activated CAFs (act CAF) also were able to induce cell death in MDA cells (Figure 7a). Furthermore, like hMSCs, CAFs were activated to express AIM2, IFIH1, TLR3, TRAIL and IFN-*β* when we activated CAFs with either poly(dA:dT) or TNF-*α* (Figure 7b) as observed in hMSCs. Such levels were increased even more when CAFs were treated with both poly(dA:dT) and TNF-*α* (Figure 4b). These results suggest that the signals of ISs, TRAIL and IFN-*β* in breast cancer sample microarrays in Figure 6 could be derived from CAFs and CAFs may have the ability to suppress tumor progression, which could be triggered by cancer cell death. Since we observed that CAFs can be activated to express TRAIL and ISs by inflammatory stimulation such as DNA fragment (poly(dA:dT)) or TNF-*α*, cancer cell death may create the feed-forward stimulation we observe here (Figure 7c).

## Discussion

In our previous study,^23, 24^ we showed that TRAIL-expressing act hMSCs induce cell death in MDA cells, and RNA/DNA released from these apoptotic DNA cells further increase TRAIL expression in act hMSCs through ISs (TLR3, AIM2 and IFIH1), resulting more cell death in MDA cells. Here, we showed that these RNA/DNA fragments also triggered AIM2 or IFIH1-mediated IFN-*β* secretion in act hMSCs, which in turn induced IRF7-mediated TRAIL expression in MDA cells. Furthermore, act hMSC-derived IFN-*β* also affected MDA cells to become less aggressive and tumorigenic (Figure 7c). In contrast, we showed that rhTRAIL-treated MDA cells became more invasive and resistance to TRAIL-induced apoptosis. It has been widely observed that cancer cells that survived after antitumor treatment become more aggressive, invasive, resistance to the treatment, which is a major challenge for cancer treatment. Indeed, it has been shown that TRAIL-sensitive cancer cells acquire TRAIL-resistant mechanisms upon exposure to the rhTRAIL^32, 33, 34^ and become an aggressive phenotype.^48^ Considering these problems, our finding is significant as it shows that activated hMSCs not only directly induce apoptosis of MDA cells but also reduce metastatic features in cancer cells. Furthermore, the paracrine effect of act hMSCs on cancer cells could lead to the new therapeutic regimen since only 24-h coculture created the long-lasting tumor-suppressive effect on MDA cells (Figures 1 and 5).

Many studies have explored IFN-*β* as a potential cancer therapy, as it reduces proliferation and inhibits tumor formation in many cancer cells.^49, 50^ However, recombinant IFN-*β* protein treatment has been unsuccessful because of their rapid degradation upon systemic delivery.^49^ To resolve such issue, several studies were performed to explore the possibilities of using carriers such as hMSCs to deliver IFN-*β* by overexpressing IFN-*β* using viral transfection.^51, 52^ Here, we show, for the first time, that hMSCs have the ability to express IFN-*β* naturally by crosstalk with cancer cells through ISs, AIM2 and IFIH1, and have a tumor-suppressive role by not only inducing apoptosis but also inhibiting migrating and invasive properties of cancer cells. The tumor-suppressive effect of hMSC-derived IFN-*β* was proven by blocking IFN-*β* activity (Figure 5). However, the reduced tumorigenic properties in MDA cells after coculture with act hMSCs were partially negated by the IFN-*β* blockage, which could be due to either (i) the reduced efficacy of the neutralizing antibody over 24 h or (ii) other antitumorigenic factors secreted from act hMSCs during coculture, such as DKK3 as we have shown previously.^23^ Nevertheless, our result showed that IFN-*β* is one of the major antitumorigenic factors secreted from act hMSCs by crosstalk with cancer cells.

Although we showed that the expression of the ISs such as AIM2 and IFIH1 was beneficial to suppress TRAIL-sensitive cancer cells, the expression of these ISs in the tumor may have a controversial effect because inflammatory responses are known to increase the risk for the development and promotion of cancer.^53^ It has been shown that activation of TLR3 in melanoma cancer cells increases their tumorigenicity and migration capacity.^54^ In contrast, expression of functional TLR3 in cancer cells reduces tumorigenicity and exhibits apoptosis.^55, 56^ Also, intra-tumoral expression of TLR3 correlate with survival and good clinical outcomes in cancer patients with hepatocellular carcinoma, with a decreased risk of metastatic relapse because of the increased T-cell and natural killer cell infiltration.^57^ In addition, AIM2 and IFIH1-mediated inflammasomes may operate at the cell autonomous level to eliminate malignant precursors through programmed cell death or, conversely, may stimulate the production of trophic factors for cancer cells and their stroma.^58^ Our analysis also showed that elevated expression levels of IFIH1 and AIM2 only correlated with good outcome in ER-negative and basal-like breast cancers, which are mainly TRAIL-sensitive TNBC.^40^ Furthermore, our results showed that the hMSCs did not increase expression of ISs and IFN-*β*, following coculture with TRAIL-resistant breast cancer cell lines, MCF-7 and BT20 cells. These results suggest that the IS-mediated feed-forward stimulation in the tumor stroma may have suppressive effects on only TRAIL-sensitive cancer cells.

A recent study showed that a stroma-related gene signature predicts clinical outcome in whole tumor samples comprising tumor epithelial cells and stroma.^22^ This study showed that the gene set associated with the poor outcome links to angiogenic, hypoxic and tumor-associated macrophage responses and the gene set associated with the good outcome links to T-helper type 1 immune responses. However, these responses of cancer microenvironment have been linked to the outcome of all types of cancers.^59, 60, 61, 62^ In contrast, our results show that the crosstalk signal genes between act hMSCs and MDA cells accurately predicted outcome in ER-negative and basal-like breast cancers, which are mainly TRAIL-sensitive cancer cells. We also observed that CAFs exhibited functional similarities to hMSCs, such as upregulated expression of TRAIL and IFN-*β* upon synthetic DNA stimulation. Considering such similarities between hMSCs and CAFs and strong evidences showing transition of hMSCs into CAFs in tumor microenvironment, we speculate that the initial cancer cell death induced by chemotherapy or radiation may cause a feed-forward stimulation in the CAFs and thereby the CAFs can suppress tumor progression or metastasis by upregulating expression levels of TRAIL and IFN-*β* in TRAIL-sensitive breast cancer patients. Furthermore, the crosstalk signals between cancer cells and stromal cells may help to predict the responses of cancer microenvironment to TRAIL-sensitive cancer cells and even identify patient groups that will receive the greatest benefits from TRAIL or IFN-*β*-based therapies.

In summary, our results suggest that the crosstalk between TRAIL-sensitive cancer cells and stromal cells creates tumor-suppressive microenvironments and further provide a novel therapeutic approach to target stromal cells within cancer for TRAIL sensitive cancer treatment.

## Materials and Methods

### Cell preparations

hMSCs and MDA cells were prepared as previously described.^23^ Hcc38, MCF-7 and BT20 cells were purchased from ATCC (Manassas, VA, USA) and cultured as suggested by the manufacturer. Cells were passaged 1 : 3 when 70% confluent. CAFs were purchased from Asterand Bioscience (Detroit, MI, USA), and cultured using *α*-MEM containing 16% fetal bovine serum (FBS; lot-selected for rapid growth of hMSCs; Atlanta Biologicals, Inc., Norcross, GA, USA), 100 units/ml penicillin, 100 *μ*g/ml streptomycin and 2 mM l-glutamine (Life Technologies, Carlsbad, CA, USA). Cells were passaged 1 : 3 when 70% confluent. Passages 2 to 3 of hMSCs and passages 4 to 5 of CAF were used for all experiments.

### Magnetic-activated cell sorting

hMSCs and cancer cells (MDA or MCF-7 cells) were cocultured as previously described.^23^ Briefly, hMSCs (1 × 10^5^ cells) and cancer cells (1 × 10^5^ cells) were plated in a six-well plate with or without TNF-*α* (20 ng/ml). After 24 h of coculture, the cells were labeled with CD90-PE (clone Thy1/310; Beckman Coulter, Brea, CA, USA) for 30 min at 4 °C and anti-PE MicroBeads (Miltenyl Biotec, Bergisch Gladbach, Germany) for 30 min at 4 °C and separated using LS (positive separation for hMSCs) or LD (negative separations for cancer cells) columns on QuadroMACS separator (Miltenyl Biotec) according to the manufacturer's protocol. The purity of sorted cells was checked using flow cytometry (Cytomics FC500; Beckman Coulter).

### Flow cytometry analysis

hMSCs and MDA cells from coculture were labeled with anti-CD90-PE (clone Thy1/310; Beckman Coulter) and CD44-PE/Cy5 (clone G44-26; BD Biosciences) for 45 min on ice, washed with PBS by centrifugation and analyzed using flow cytometry.

### Cell migration assay

*In vitro* cell migration was examined using Transwell culture inserts (BD Biosciences, Franklin Lakes, NJ, USA) with 8 *μ*m pore filter inserts for 24-well plates. Briefly, 5 × 10^5^ cells suspended in serum-free DMEM were added to the inserts, the upper chamber, which was placed in a 24-well culture plate. FBS was added to the lower chamber at the final concentration of 2% as a chemoattractant. After 6 h, the upper insert was removed, washed and the non-filtered cells were gently removed with a cotton swab. Filtered cells located on the lower side of the chamber were stained with crystal violet, photographed and counted using Image J ver.1.48 (http://imagej.nih.gov/ij/).

### Cell invasion assay

*In vitro* cell invasion assay was performed using Cultrex 96 Well 3D Spheroid BME Cell Invasion kit (Trevigen, Inc., Gaithersburg, MD, USA) according to the manufacturer's protocol. Briefly, 3 000 cells resuspended in spheroid formation ECM were dispensed in a well of the 3D Culture Qualified 96 Well Spheroid Formation Plate. The plate was then spun down and incubated at 37 °C for spheroid formation. After 24 h, invasion matrix was added and incubated at 37 °C. At chosen time points, images of invading spheroids were taken, and the total area of invading spheroid was calculated with Image J.

### *In vivo* tumorigenicity and metastasis assay

Either control MDA cells (1 × 10^4^ or 1 × 10^5^ in 100 *μ*l of HBSS) or MDA cells that were isolated from coculture with activated hMSCs and IgG or IFN-*β*-neutralizing antibody were inoculated into mammary fat pad of 6-week-old female NOD/SCID mice. After the single injection of MDA cells, mice were palpated for tumor growth weekly after tumor implantation. Once palpable, tumors were measured in two dimensions (length and width) using a digital caliper to calculate the volume. After 4–6 weeks from cell inoculation, all mice were killed, and tumors in the fat pad were collected, and tumor volumes were measured. To examine metastasis, lungs were collected for genomic DNA isolation to detect hAlu sequences using real-time PCR.^23^

### Animals

Six-week-old female NOD/SCID mice (NOD.CB17-Prkdcscid/J) from the Jackson Laboratory (Bar Harbor, ME, USA) were used under a protocol approved by the Institutional Animal Care and Use Committee of Texas A&M Health Science Center College of Medicine.

### Genomic DNA extraction/real-time PCR assays for human Alu sequences

Genomic DNA from lung samples was extracted, and real-time PCR was performed to detect human Alu signals, as previously described.^28^

### Preparation of apoptotic MDA cells

MDA cells were plated in serum-free *α*-MEM with 100 ng/ml rhTRAIL (R&D Systems, Minneapolis, MN, USA). After 24 h at 37 °C, floating cells were collected and washed by centrifugation at 500 x *g* for 5 min. The pellet was resuspended in 2% culture medium (2% CM; alpha-MEM containing 2% FBS, 100 units/ml penicillin, 100 *μ*g/ml streptomycin and 2 mM l-glutamine) and plated on hMSC containing wells. For RNase and DNase treatment, apoptotic MDA cells were washed by centrifugation, resuspended in 0.2 ml PBS containing either 20 *μ*g of RNase (QIAGEN, Valencia, CA, USA) or 30 units of DNase (QIAGEN), and incubated for 2 h at 37 °C. The cells were washed by centrifugation and resuspended in 2% CM before adding to hMSC containing wells.

### Cell death assay

hMSCs and MDA cells from 24-h coculture experiments with or without TNF-*α* (20 ng/ml) were labeled with CD90 and incubated at room temperature for 20 min followed by 300 ng/ml Annexin V (Annexin V-FITC Apoptosis Detection Kit; Sigma-Aldrich, St. Louis, MO, USA) and 4 *μ*g/ml 7-aminoactinomycin D (Sigma-Aldrich) to detect apoptotic cells. The labeled cells were analyzed by flow cytometry for cell death assay.

### RNA extraction from cultured cells and real-time RT-PCR analysis

hMSCs, MDA, Hcc38, MCF-7 and BT20 cells were separated by either MACS LS or LD columns after coculture. RNA was extracted using RNeasy Mini Kit (QIAGEN). Real-time RT-PCR analyses were performed as previously described.^23^ List of primers and probes is in the Supplementary Information.

### Transfections with siRNA

hMSCs (5 × 10^4^ cells per well in six-well plate) were transfected by incubating 4 h with 20 nM siRNA for AIM2, IFIH1 or scrambled siRNA A (Santa Cruz Biotechnology, Dallas, TX, USA) using Lipofectamine RNAiMax reagent (Life Technologies). Following transfection, the cells were recovered with 16% FBS containing *α*-MEM for 2–4 h and then cocultured with MDA cells. Duplicate wells were also prepared to check the transfection efficiency using real-time RT-PCR assay.

### Western blot analysis

Western blot analyses were performed after sorting MDA cells or hMSCs from coculture, as described above. Procedures for western blot analyses were described previously.^23^ Antibodies used in this study are listed in Supplementary Information.

### Luciferase assay

MDA cells that were stably transfected with pGL4 reporting vector (Promega, Madison, WI, USA) containing TRAIL promoter (see Supplementary Information for plasmid construction and stable transfection) were cocultured with hMSCs (10 000 cells per well in 96-well plate) with or without TNF-*α*. After 24 h, luciferase activity was detected to read TRAIL expression levels using Bright-Glo Luciferase Assay System (Promega) according to the manufacturer's protocol. In parallel, these MDA cells were cocultured with hMSCs in a six-well plate for apoptosis assay. Luciferase activity levels were normalized to the percentage of live MDA cells.

## Abbreviations

MSCs: mesenchymal stem/stromal cells
hMSCs: human MSCs
TRAIL: tumor necrosis factor-related apoptosis-inducing ligand
rhTRAIL: recombinant human TRAIL
IFN-*β*: interferon-beta
rhIFN-*β*: recombinant human IFN-*β*
TNBC: triple-negative breast cancer
MDA: MDA-MB-231
ER: estrogen receptor
CAF: cancer-associated fibroblasts
act hMSCs: hMSCs activated with TNF-*α*
CC: coculture of cancer cells with naive hMSCs
CCT: coculture of cancer cells with hMSCs and TNF-*α*
poly(dA:dT): poly(deoxyadenylic-deoxythymidylic) acid
poly(I:C): polyinosinic:polycytidylic acid
PKC-*α*: protein kinase C-alpha
TLR3: Toll-like receptor 3
AIM2: absent in melanoma 2
IFIH1: interferon induced with helicase C domain 1
IRF: interferon-related factor
IFNB1: IFN-*β*
IS: innate sensor
CM: culture medium
sup: supernatant collected from cultured cells
FBS: fetal bovine serum
IgG: IgG control antibody
