# Supplementary material for: Activated human mesenchymal stem/stromal cells suppress metastatic features of MDA-MB-231 cells by secreting IFN-β
Source: Cell Death Dis. 2016 Apr 14;7(4):e2191–. doi: 10.1038/cddis.2016.90 (PMC4855669; doi:10.1038/cddis.2016.90)
Supplement: Supplementary Information [file cddis201690x1.docx]

**Supplemental Information**

**Figure S1. MDA separation from coculture with hMSCs using MACS.** Flow cytometry analysis on (a) coculture of hMSC and MDA cells and on (b) MDA cells after negative sorting of CD90 positive hMSCs using MACS (Magnetic activated cell sorting).

**Figure S2.** **Expression of IFN-β in hMSCs after stimulation of poly (dA:dT) and TNF-α.** Quantitative RT-PCR for IFN-β from MSCs treated with or without TNF-α (10 ng/ml) or poly (dA:dT) for 24 hrs. MSCs increase IFN-β1 mRNA expression upon poly (dA:dT) stimulation in dose dependent manner.

**Figure S3. Assessment of the efficacy of AIM2 siRNA and IFIH1 siRNA in hMSCs.** Quantitative RT-PCR assays for AIM2 (A) and IFIH1 (B) in the hMSCs that were isolated from the experiment in Figure 4D.

**A B**

**Figure S4. IFN-β upregulates TRAIL expression in hMSCs during coculture with MDA cells.** (A~D) Real-time RT-PCR assays for AIM2, IFIH1, IRF7 and TRAIL in hMSCs treated with different concentrations of rhIFN-β. Values are mean ± S.D. for triplicate of assay. (E~H) Real-time RT-PCR assays for AIM2, IFIH1, IRF7 and TRAIL in hMSCs treated with supernatant from act hMSC-MDA coculture (CCT sup) that was treated with different concentrations of IFN-β neutralizing antibody (α-IFN-β). Values are mean ± S.D. for triplicate of assay.

**Figure S5.** **Kaplan-Meier survival analysis of the innate sensors and related genes in breast cancer.** (A-C) Kaplan-Meier survival analysis of the AIM2 (A), IFIH1 (B), TLR3 (C) in all and ER-positive breast cancer.

**List of primers**

| Name | Taqman Gene Expression Assay ID  (Life Technologies) |
| --- | --- |
| Human-specific GAPDH | Hs00266705_g1 |
| Human TRAIL (TNFSF10) | Hs00921974_m1 |
| Human TRAIL receptor 1 (DR4; TNFRSF10A) | Hs00269492_m1 |
| Human TRAIL receptor 2 (DR5; TNFRSF10B) | Hs00366278_m1 |
| Human AIM2 | Hs00915710_m1 |
| Human IFIH1 | Hs01070332_m1 |
| Human Fas | Hs00236330_m1 |
| Human Fas Ligand (FASLG) | Hs00181225_m1 |

**List of Antibodies**

| Antigen | Clone | Company | Dilution |
| --- | --- | --- | --- |
| TRAIL | Rabbit mAb (C92B9) | Cell Signaling Technology | 1:1000 |
| PKC-α | Rabbit mAb | Cell Signaling Technology | 1:1000 |
| Phospho-PKC-α | Rabbit mAb (Thr638/641) | Cell Signaling Technology | 1:1000 |
| β-actin | Clone AC-15 | Sigma Aldrich | 1:25000 |

For secondary antibodies, HRP-linked anti-rabbit IgG or anti-mouse IgG (Cell Signaling Technology; 1:2,000) were used.

**Preparation of TRAIL reporter construct.**

Genomic DNA isolated from hMSCs (QIAamp Tissue Kit; QIAGEN) was used as the template with primers for the human TRAIL promoter (Figure 1 for sequences for primers), using the Expand High Fidelity PCR System (Roche Molecular Biochemicals) following the manufacturer’s instruction. The amplified DNA fragments (1183bp) were subcloned into the *KpnI* and *NheI* sites in the pGL4 plasmid containing the luciferase reporter gene (pGL4-17; Promega). The PCR construct was amplified between the highlighted area in TRAIL promoter sequence.

1 aaaatttgaaaatattttcttaaatgtagactcatttacagatagaaggcaagggcagga

61 agtgatggtgaccagcggtgcctgaatgaactcaggaatgtaactgtagatctagggtcc

121 caaactttaggtttcaaaggatctcttggagtacttgctgaaaaatgtaggttcctaagt

181 ccactgccagaaactctgactcagtgggtcaagaatggaataactaaacaatggccccat

241 gcagtggttcatgcctgtaatcccagcacgttgggaggttgaagcaagaggatcacttga

301 ggtcaggagttcgagaccagcctggcctacatgataaaaccccatctctactaaaaatac

361 aaaaaaattagctgggcatggtggcatgcacctgtaatcccagctacttgggaggctgag

421 gcaggagaattgcttgaatctgggaggtggaggttgtagtgggccgagattgtgccattg

481 caccactgcactccagcctgggcgataaagtgagattctgtcaaaaaaataaataaatac

541 atgaaagagagaaagaaagaaagaaagaaagaaagaaagaaagaaagaaagaaagaaaga

601 aagaaagaaagaaggaaagaaggaaagaatagaaaagaaaagaaagaaagggaggaagaa

661 aaggaaagaaagaaatgctgaataagatatagagacacatacagctgggccagcttatga

721 catctgatagtggggagatttggggctgggtcctgaatctgagggtaattaactccctgt

781 aacttcttttcctaatctgtaaaaggatagtgacagcgagacattgtgatggggttaata

841 ttttggaaaacatccacatgtttttttcctttgcctttctgagtgtgtcaactacttcct

901 acctgtccagcctaacacacaggcatattgtcttggtagggatggagatctgagaaggag

961 attagaatttgtgtctgaaggtttgcaaagaggaagaagtcgtcaatatttagattctga

1021 cattcaagatggaattatgtagcaagaccattgctatgagacagtatttctattttcctt

1081 tatccactcccaccctgccctcttcccaccctcacagtagcatgagaaaaaccacatatg

1141 gaagtttcaggtcataaaaattatcttataatttagaaaacaggccttgtgcctatgaca

1201 gccaggccatgaggcttagagctctgtggtagaatgaggatatgttagggaaaagcaaag

1261 aaaatccctcccctccttggctgaggacattatcaaaaggagagcaagaaagagaagaga

1321 gaaatgggcttgaggtgagtgcagataaggggtgcatggatcctgagggcaaggagagga

1381 gcttctttcagtttccctcctttccaacgactactttgagacaagagctgtccctgggca

1441 gtaggaagggggagggacagttgcaggttcaatagatgtgggtggggccaaggccacaga

1501 acccagaaaaacaactcattcgctttcatttcctcactgactataaaagaatagagaagg

1561 aagggcttcagtgaccggctgcctggctgacttacagcagtcagactctgacaggatcat

1621 ggctatgatggaggtccaggggggacccagcctgggacagacctgcgtgctga

**Stable transfection**

MDA cells were transfected with TRAIL-pGL4 reporter using Lipofectamine 2000 (Life Technologies) as manufacturer’s instruction. After 72 hours of transfection, MDA cells were treated with 1000 μg/mL neomycin (Sigma) for the first week and 500 μg/mL neomycin in DMEM containing 10 % FBS for 2 more weeks. The media was changed every 3^rd^ day and the cells were passaged in 1:4 upon 80 % confluency. The transfected cells were than plated <100 cells/cm^2^ and cultured until the cells start to form colonies. The colonies were then isolated and cultured until they reach sufficient number. Expanded clone of MDA cells were tested for luciferase activity and the clone with the strongest activity was selected for further experiments
